# Estimating Oxygen Needs for Childhood Pneumonia in Developing Country Health Systems: A New Model for Expecting the Unexpected

**DOI:** 10.1371/journal.pone.0089872

**Published:** 2014-02-20

**Authors:** Beverly D. Bradley, Stephen R. C. Howie, Timothy C. Y. Chan, Yu-Ling Cheng

**Affiliations:** 1 Centre for Global Engineering, University of Toronto, Toronto, Canada; 2 Department of Chemical Engineering and Applied Chemistry, University of Toronto, Toronto, Canada; 3 Child Survival Theme, Medical Research Council Unit, The Gambia, Banjul, The Gambia; 4 Department of Mechanical and Industrial Engineering, University of Toronto, Toronto, Canada; University of Liverpool, United Kingdom

## Abstract

**Background:**

Planning for the reliable and cost-effective supply of a health service commodity such as medical oxygen requires an understanding of the dynamic need or ‘demand’ for the commodity over time. In developing country health systems, however, collecting longitudinal clinical data for forecasting purposes is very difficult. Furthermore, approaches to estimating demand for supplies based on annual averages can underestimate demand some of the time by missing temporal variability.

**Methods:**

A discrete event simulation model was developed to estimate variable demand for a health service commodity using the important example of medical oxygen for childhood pneumonia. The model is based on five key factors affecting oxygen demand: annual pneumonia admission rate, hypoxaemia prevalence, degree of seasonality, treatment duration, and oxygen flow rate. These parameters were varied over a wide range of values to generate simulation results for different settings. Total oxygen volume, peak patient load, and hours spent above average-based demand estimates were computed for both low and high seasons.

**Findings:**

Oxygen demand estimates based on annual average values of demand factors can often severely underestimate actual demand. For scenarios with high hypoxaemia prevalence and degree of seasonality, demand can exceed average levels up to 68% of the time. Even for typical scenarios, demand may exceed three times the average level for several hours per day. Peak patient load is sensitive to hypoxaemia prevalence, whereas time spent at such peak loads is strongly influenced by degree of seasonality.

**Conclusion:**

A theoretical study is presented whereby a simulation approach to estimating oxygen demand is used to better capture temporal variability compared to standard average-based approaches. This approach provides better grounds for health service planning, including decision-making around technologies for oxygen delivery. Beyond oxygen, this approach is widely applicable to other areas of resource and technology planning in developing country health systems.

## Introduction

Pneumonia is the leading cause of child mortality globally, representing 18% of the 7 million deaths under-five in 2011 [1]. Pneumonia is most prevalent during the rainy season in tropical regions, and during the cooler, drier winter season elsewhere [2]–[10]. Pneumonia is associated with severe hypoxaemia (arterial blood oxygen saturation, SpO_2_, below 90%), a potentially fatal complication that requires oxygen therapy [11]–[13].

Medical oxygen is an important example of a health commodity that is not widely or reliably available in many low-income settings due to financial constraints, poor infrastructure (e.g., roads, electricity), and inadequate capacity for supply management and equipment maintenance [14]–[16]. Access to oxygen has been found to be particularly inadequate in paediatric wards due to insufficient supply and competition for use by other services [17]. Anecdotally, oxygen is often rationed to only the sickest children during busy periods [18]. The World Health Organization (WHO) even offers suggestions on how to prioritize the use of limited oxygen supplies [19]. Given that oxygen has been shown to reduce mortality from pneumonia in children by as much as 35% [20] improved oxygen supply to this patient population has the potential to substantially reduce child deaths.

To plan for reliable and cost-effective supply, the paediatric oxygen needs of a health facility must be understood, but collecting longitudinal clinical data is difficult in developing countries. One approach to overcoming this challenge is to periodically assess the supply/demand mismatch over a short time period (e.g. 24-hours [21]), but this does not capture oxygen shortages throughout the year nor provide insight into how to effectively adjust the supply to meet changing needs. Another approach is to project oxygen demand using average estimates of key factors (e.g., annual admission rate, treatment duration, flow rate, etc.) [22], [23]. However, this approach will underestimate oxygen demand a large proportion of the time because it does not consider peaks arising from: (a) seasonal variations in respiratory disease burden and the corresponding disproportionate need for oxygen, and; (b) random variations in patient-specific factors that occur on shorter time scales.

Given the difficulties in collecting long-term demand data, and the shortcomings of using averages, the objective of this paper is to present a discrete event simulation (DES) model for estimating demand for a seasonal health commodity, using the example of oxygen for childhood pneumonia. DES is a well-accepted computer simulation technique in health services research, particularly in the assessment and design of health care delivery systems, and in forecasting demand for human and physical resources [24]–[27]. However, the application of DES to study health service delivery challenges in low-income countries is still in its infancy. Only recently have models been developed to evaluate the cost-effectiveness of new technologies or interventions [28]–[30], or the impacts of policy changes affecting service delivery operations [31] or supply chain logistics [32], [33]. To our knowledge, forecasting temporal demand for a health commodity used in a critical care in-patient environment is a novel application of DES for low-income health systems.

We hypothesize that DES will provide realistic time-varying oxygen demand estimates, and will allow for the first time the ability to quantify temporal variations in simultaneous demand, expressed as either patient load or oxygen flow rate, at a health facility level. Oxygen demand due to childhood pneumonia is dependent on seasonality, annual case load, hypoxaemia prevalence, and variations in individual patients’ prescribed flow rates and treatment durations. These key demand factors underpin our model and are described further below.

### Factors Affecting Oxygen Demand

#### Seasonality

Pneumonia among children is seasonal [8]–[10], [34]–[37]. In the pneumonia “high season”, which typically lasts 3 to 5 months, the average monthly case load can be 20 to 90% greater than that of the “low season”, depending on the degree of seasonality (i.e., the proportion of total annual cases accounted for in high season) [2], [10], [34], [37], [38]. This seasonal difference in case burden is primarily due to outbreaks of viral pathogens, such as respiratory syncytial virus, during hot, rainy months in tropical regions [2]–[5], [38], [39], and outbreaks of influenza viruses during cooler, drier months in more temperate regions [6].

#### Pneumonia case load

Although global estimates of pneumonia incidence and mortality are available [40]–[42], empirical data on admission rates for individual health facilities in low-income countries are scarce. A few studies have reported average annual pneumonia admissions ranging from about 50 cases at small rural health centres to over 1000 cases at district or main referral hospitals [14], [23], [43].

#### Hypoxaemia prevalence

Hypoxemia prevalence among childhood pneumonia cases varies widely between geographic regions and at different altitudes, as well as with pneumonia severity [11]–[13], [37]. An estimated 13.3% (IQR 9.3% - 37.5%) of WHO-defined pneumonia cases globally are hypoxaemic [12]; in lower-lying African countries, prevalence ranges from 3 to 10%, whereas in Asia at higher altitudes prevalence ranges from 9% to 39%.

#### Flow rate

The WHO-recommended flow rates when using nasal prongs are 0.5 L/min for young infants and 1 to 2 L/min for preschool aged children, with a maximum of 4 L/min [19], [44]. On average, flow rates of 0.6 to 1.0 L/min are required to achieve >90% SpO_2_, with high inter-patient variability [45]–[47]. In practice, patients will often receive equal flow rates from a source split equally among multiple patients [23].

#### Treatment duration

The duration of oxygen therapy typically ranges from 2 to 5 days [19]. A study from The Gambia found mean treatment duration for children to be 3.65±2.92 days [45]. Constant treatment durations of 3 and 2.8 days per patient were used to estimate oxygen demand in The Gambia [22] and Papua New Guinea [23], respectively, without considering variability.

## Methods

### Re-interpreting Demand Factors for a DES Model: Input Parameters and Assumptions

#### Seasonality

We model pneumonia seasonality with a single high season once per year. The proportion of annual pneumonia cases concentrated in high season – or ‘degree of seasonality’ - is a fixed percentage, *P.* The high season duration, *D*, is a fixed value in months.

#### Pneumonia case load

Pneumonia case load is modeled using random patient arrivals following a Poisson Process [48]–[50] with rate parameter, λ, which denotes the number of pneumonia arrivals per year. The *average* monthly admission rate in high season is *Pλ*/*D*. To smooth the admission profile between seasons, we assume one-month ramp up and ramp down periods as part of the high season, with monthly admission rates adjusted appropriately to reflect this profile while maintaining the prescribed high season average. In low season, the monthly admission rate is (1−*P*)*λ*/(12−*D*) and does not vary by month.

#### Hypoxaemia prevalence

Although a range of values have been reported for hypoxaemia prevalence among pneumonia cases, *H*, no data is available regarding seasonal variability. We therefore assume *H* to be invariant across seasons. The number of hypoxaemic cases, however, will vary seasonally since pneumonia incidence is seasonal.

#### Flow rate

Prescribed flow rate, *F*, is modeled as a random variable based on a modified Poisson distribution with a mean, *ρ*, of 1 L/min, a minimum value of 0.5 L/min, and discrete allowable values in increments of 0.5 L/min. This distribution reflects the WHO recommendations for infants and children [19], [44] and the reality that most patients likely receive 0.5 L/min or 1 L/min due to flow-splitting technology limitations [23]. The same distribution is used for high and low seasons.

#### Treatment duration

An exponential distribution with a mean, *μ*, of 3.5 days is used to describe the random treatment duration, *T*. An exponential distribution is a special case of the Weibull distribution, which is widely used to model ‘length of stay’ in health services [51], [52].

### Model Mechanics and Output

**Figure 1:**
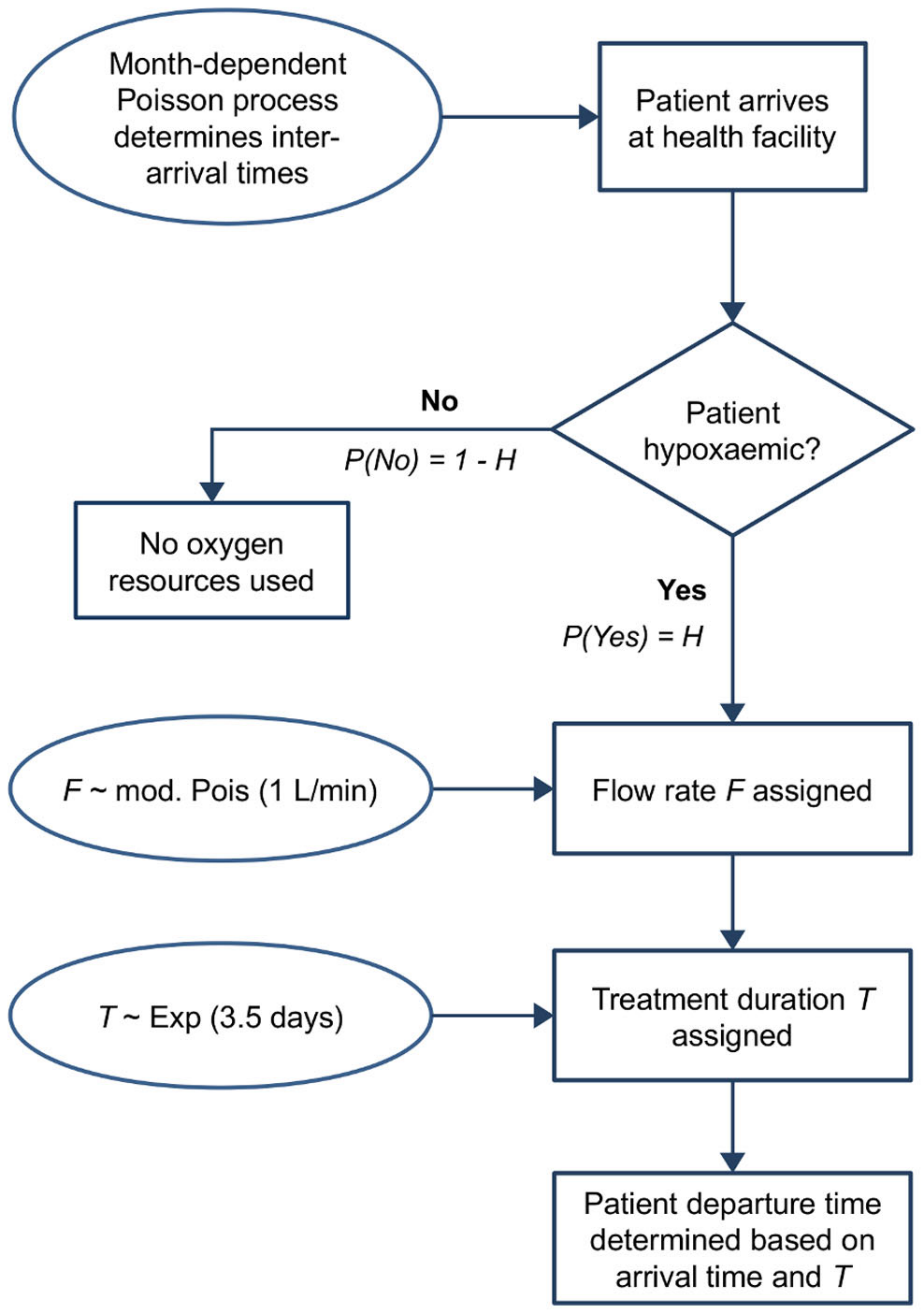
Process flow diagram of a patient’s pathway through the simulation. Simulation continues until 365 days are reached.

The DES model was developed in Matlab (MathWorks Inc., Natick, MA). Events are simulated over a one-year period, beginning with the first day of low season. Each arriving patient generated by the Poisson Process is randomly assigned to a state of hypoxaemic (needs oxygen) with probability *H*; hypoxaemic patients are further randomly assigned a flow rate and a treatment duration according to the distributions described earlier (Figure 1). At the end of the 365-day simulation period, any remaining treatment time for patients in the system are wrapped around to the beginning of the simulation year.

**Figure 2:**
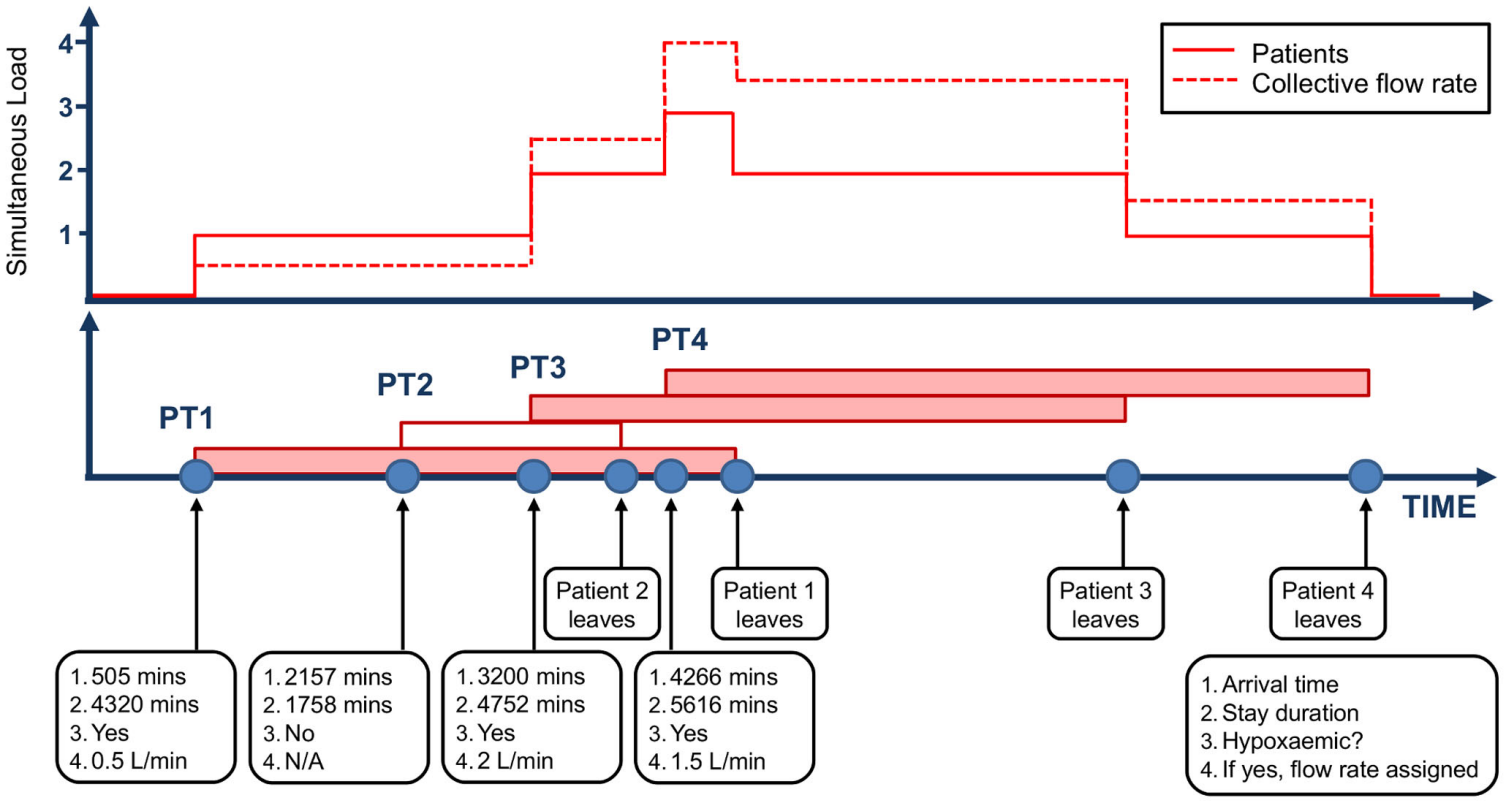
Example timeline view of simulated patient arrivals and variable assignments. Lower portion shows simulation ‘events’. Upper portion shows changing level of simultaneous patients on oxygen and collective flow rate (L/min) over time.

Simulation mechanics are illustrated in Figure 2. Random arrival times result in patients being present simultaneously for random periods of time. The number of simultaneous patients requiring oxygen and their collective flow rate vary in an un-correlated fashion because oxygen requirements differ from patient to patient.

The simulation output is aggregated into an hour-by-hour account of patient load and collective flow rate. The output can be further analyzed to determine total demand, variability in demand, or maximum peaks in demand, for any time scale (e.g., daily, monthly, seasonally, annually). These metrics are then analysed across the desired number of simulation iterations.

### Scenario Analysis

Three scenarios were selected to represent a wide range of health facilities. Input parameters for all scenarios are summarized in Table 1. 500 iterations were conducted for each set of conditions.

**Table 1:** Input parameters for modeled scenarios.

Factor	Parameter	Scenario 1	Scenario 2	Scenario 3	References
**Annual pneumonia case load**	*λ* (patients/year)	500	50 to 2,000	500	[14], [23], [43]
**Hypoxaemia prevalence**	*H* (%)	13	13	10, 20, 30	[12]
**Seasonality**	*P* (%)	45	45	35, 45, 55	[2], [10], [34], [37], [38]
	*D* (months)	4	4	4	[10], [34]–[37]
**Flow rate**	*F* (L/min)*	*ρ* = 1	*ρ* = 1	*ρ* = 1	[19], [23], [44] ‡
**Treatment Duration**	*T* (days) †	μ = 3.5	μ = 3.5	μ = 3.5	[19], [22], [45] ‡

*random variable with modified Poisson distribution;

† random variable with exponential distribution;

‡ references support parameter value selection, not the type of distribution chosen to describe the demand factor.

Scenario 1 illustrates the implications of considering demand on an hourly basis by visually comparing DES output for a typical setting with estimates from an average-based approach characterized by the same annual case load and hypoxaemia prevalence, but no seasonal variation, and constant (average) values for flow rate and treatment duration.

Using the same hypoxaemia prevalence (*H*), degree of seasonality (*P*), and high season duration (*D*) as Scenario 1, annual pneumonia case load (*λ*) was varied from 50 to 2,000 in Scenario 2. Total oxygen demand was computed for both high and low seasons and compared to average-based demand estimates.

For Scenario 3, *H* and *P* were varied to explore the effects of these context-specific factors on peak demand. Three levels of *H* (10%, 20% and 30%) covering the interquartile range of the global systematic review [12], and three levels of *P* (35%, 45% and 55%) were selected, giving nine combinations of these two parameters. Note that for a 4-month high season, *P* = 35% represents a very low degree of seasonality (i.e., 35% of cases in 33 ⅓ % of the year). We analyzed ‘peak demand’ in terms of both *patients* and *time*. First, we found the maximum simultaneous *patient load* in each season. Then, we computed the amount of *time* spent at or above selected peak patient load thresholds, as well as the amount of *time* that demand (collective flow rate) exceeded average-based estimates.

## Results

### Model Verification

All input parameters for Scenario 1 fell within the 95% confidence intervals of the corresponding simulated outcomes (Table 2).

**Table 2:** Verification of model output for Scenario 1.

Factor	Parameter	Scenario 1 Input	Simulation Outcome (Mean [95% CI])
**Annual pneumonia case load**	*λ* (patients/year)	500	500.5 [498.5, 502.5]
**Hypoxaemia prevalence**	*H* (%)	13	13.1 [12.9, 13.2]
**Seasonality**	*P* (%)	45	44.8 [44.6, 45.0]
	*D* (months)	4	4
**Flow rate**	*F* (L/min)	*ρ* = 1	1.00 [0.99, 1.00]
**Treatment Duration**	*T* (days)	μ = 3.5	3.51 [3.47, 3.54]

### Scenario Results

#### Scenario 1

**Figure 3:**
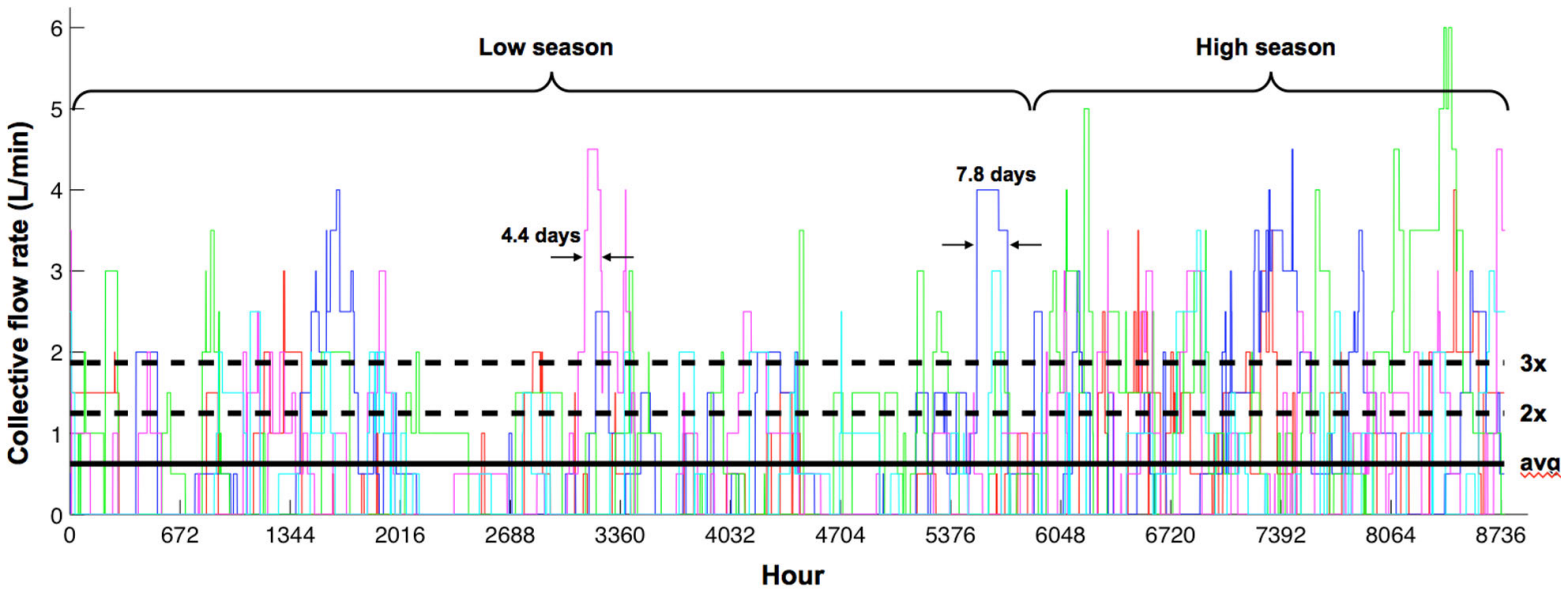
Hourly oxygen demand for a typical health facility. Scenario inputs were 500 pneumonia admissions per year (*λ*), degree of seasonality (*P*) of 45%, high season duration (*D*) of 4 months, and hypoxaemia prevalence (*H*) of 13%. Different coloured lines represent five distinct simulation iterations. Horizontal lines represent the average-based estimate (solid), as well as 2 and 3 times this estimate (dashed), for this particular scenario. Prolonged periods of 4.4 and 7.8 days exceeding 3 times the average level in low season are shown.

Figure 3 illustrates how hourly oxygen demand fluctuates throughout the year due to seasonality and variability in patient arrivals, treatment duration, and flow rate. Only five out of the 500 iterations (i.e. simulated years) of Scenario 1 are shown. Constant flow rate demand corresponding to an average-based estimate, as well as double (2X) and triple (3X) this average estimate, are also shown. Simulated demand exceeds the constant average-based estimate 29.3% and 43.8% of the time in low and high season, respectively. Demand often exceeds the 3X level for several consecutive days, even in low season (Figure 3).

#### Scenario 2

**Figure 4:**
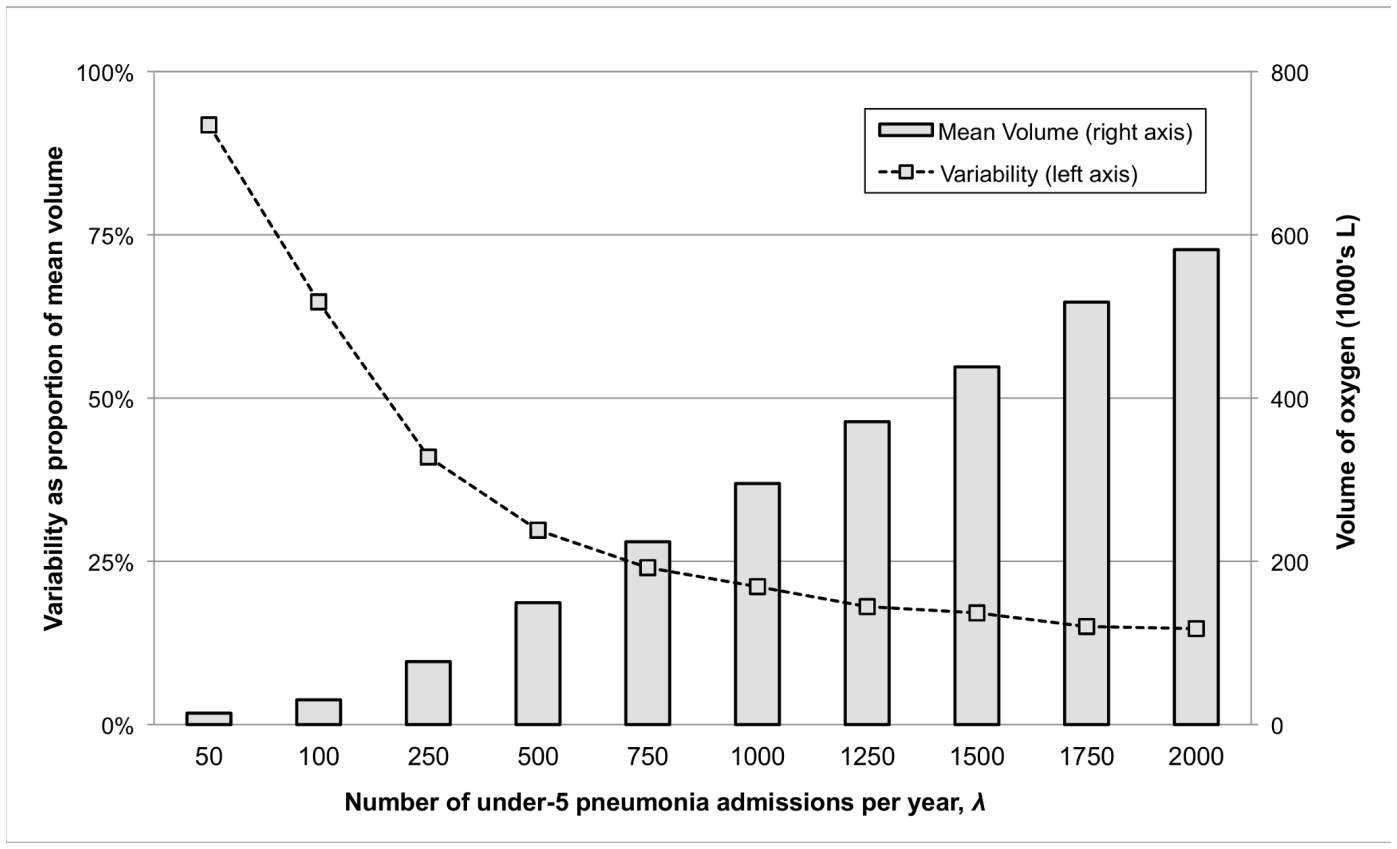
Total oxygen demand and year-to-year variability in high season for pneumonia admissions (*λ*) ranging from 50 to 2000. Other scenario inputs: degree of seasonality (*P*) of 45%, high season duration (*D*) of 4 months, and hypoxaemia prevalence (*H*) of 13%. Mean high season volume is averaged across 500 simulation iterations (right axis). Standard deviation as a percentage of the mean high season volume is plotted to represent variability across simulation iterations (left axis).

In Scenario 2, oxygen demand increases with increasing annual pneumonia case load (*λ*), as expected (Figure 4). When aggregated as annual totals, simulated estimates match closely with average-based estimates. However, the DES approach allows for the calculation of seasonal totals as well as year-to-year variability from multiple iterations. For example, the standard deviation of annual high-season oxygen demand across iterations ranges from about 15% of the mean high season volume for *λ* = 2,000 to over 90% for *λ* = 50, suggesting that smaller health centres have much less predictable oxygen needs from year to year (Figure 4). Trends were identical for low season (figure omitted).

#### Scenario 3

**Figure 5:**
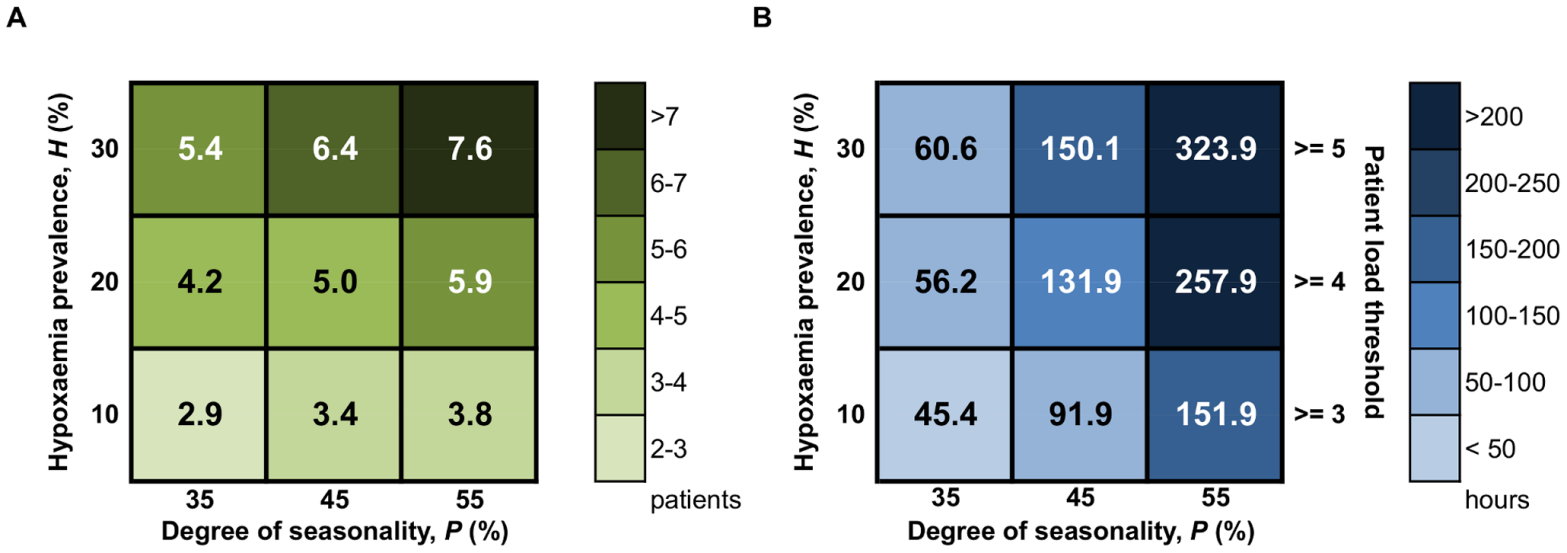
Peak demand in high season, measured in terms of both patients and time. Sensitivity matrices show (A) maximum simultaneous patient load in high season; and (B) amount of time (hours) patient load exceeds selected peak patient load thresholds in high season, for hypoxaemia prevalence (*H*) ranging from 10 to 30% and degree of seasonality (*P*) ranging from 35 to 55%, averaged across 500 simulation iterations. In (B) thresholds of 3, 4 and 5 simultaneous patients were used for *H* levels of 10%, 20%, and 30%, respectively. Results are for *λ* = 500 pneumonia admissions per year.

**Figure 6:**
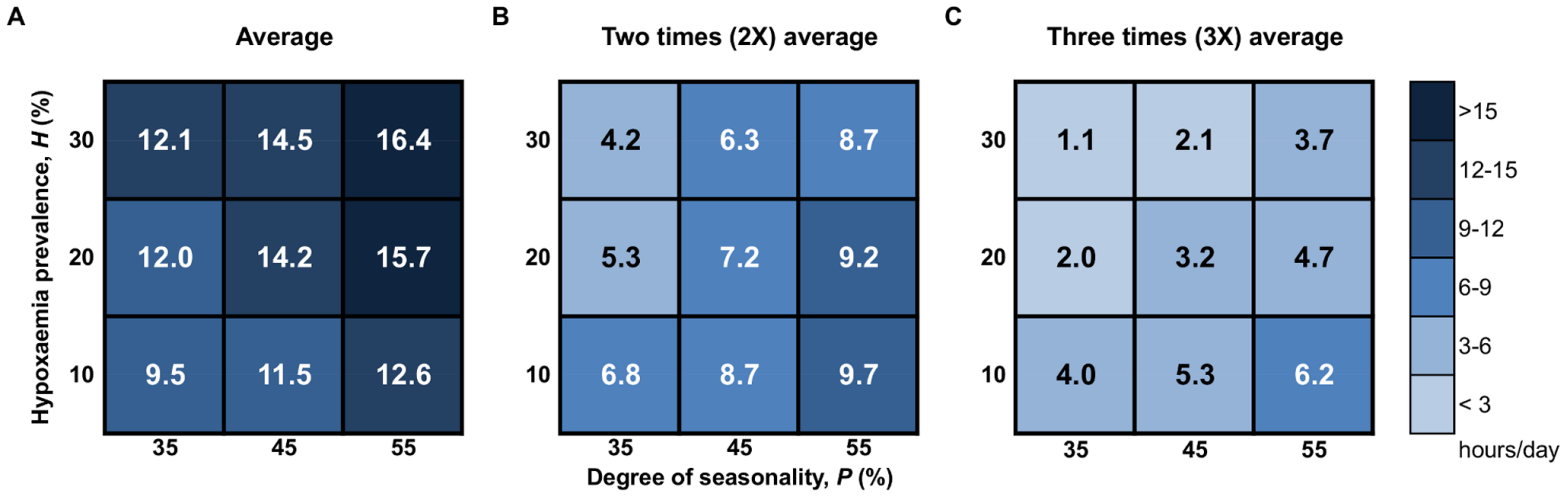
Peak demand in high season, measured in terms of time above average level thresholds. Sensitivity matrices show amount of time (hours/day) in high season that oxygen demand exceeds (A) 1 times; (B) 2 times, and; (C) 3 times average-based demand estimates for hypoxaemia prevalence (*H*) ranging from 10 to 30% and degree of seasonality (*P*) ranging from 35 to 55%, averaged across 500 simulation iterations. Results are for *λ* = 500 pneumonia admissions per year.

Results from Scenario 3 on the effects of hypoxaemia prevalence and degree of seasonality are shown in Figures 5 and 6. Figure 5a shows a sensitivity matrix of maximum simultaneous patient load expected in high season. Maximum patient load nearly doubles as *H* increases from 10% to 30%, as shown by the sharp gradient from bottom to top. Maximum patient load also increases with *P*, but with a weaker dependence than *H*. Similar observations were seen for low season (figure omitted).

The duration of time that patient load is at or above selected thresholds – representing possible health facility capacity limits – is also an important consideration. *P* strongly influences the time spent at or above peak patient load thresholds during high season, as shown by the sharp gradient from left to right in Figure 5b. For example, the amount of time at or above five patients is five times greater where *P* = 55%, compared to where *P* = 35% (i.e., very little seasonality). Conversely in low season, the amount of time above peak patient thresholds is greater when *P* is *lower*.

Figure 6 shows peak demand in terms of the amount of time that simulated demand exceeded average-based estimates, and double and triple these estimates. With increasing *H* and *P*, oxygen demand exceeded average-based estimates anywhere from 9.5 to 16.4 hours per day in high season. In low season, demand exceeded the average between 6.7 and 11.4 hours per day (low season results not shown in figure).

As *H* increases, the 2X and 3X estimates increasingly encompass true variability in demand, as shown by the darkening gradient from top to bottom in Figure 6b and 6c. For example, at *H* = 10%, demand exceeds the 3X estimate by as much as 3.6 to 6.2 hours per day in low and high season, respectively; while the corresponding values when *H* = 30% are only 0.9 and 3.7 hours, respectively (low season results not shown in figure). We hypothesize that with lower *H*, the flow rate and treatment duration are more dominant sources of variability, causing demand to fluctuate widely around lower constant average-based demand estimates.

## Discussion

We present a model for estimating oxygen demand due to childhood pneumonia, which leverages the distinctive time-based approach of DES in order to capture temporal variability in key demand factors. Modeling results for a range of hypothetical low-resource settings reveal that substantial year-to-year variability in oxygen demand can exist, particularly for small health facilities. We also show that average-based estimates can severely underestimate demand during seasonal highs as well as random peaks throughout the year; for as many as 16 hours per day in high season, and as many as 11 hours per day in low season. This means that with a system tailored to meet average demand levels, oxygen shortages may be experienced up to 68% of the time in high season, and about half the time in low season, leaving many patients under-served or even untreated altogether.

Our approach also enables the analysis of sensitivity to different demand factors; such inter-dependent relationships can help inform oxygen supply planning. For example, maximum patient load was more dependent on hypoxaemia prevalence, whereas time at or above peak demand loads was more dependent on degree of seasonality. Maximum patient load has implications for the physical technology capacity needed to meet demand (e.g., number of oxygen concentrators, flow-splitting devices, nasal prongs, etc.), whereas time spent at certain demand levels has implications for more temporally-based supply management and planning issues, both financial and logistical (e.g., cost per kWh to operate concentrators and/or generators, cylinder depletion rates and refilling frequency, etc.). Thus, to accommodate peak patient loads, it might be more important to plan around hypoxaemia prevalence, whereas to accommodate sustained time at peak loads, the degree of seasonality would be the foremost factor to consider. Our approach also allows for the analysis of how such inter-dependent relationships might differ between low and high season, which could inform better temporally-based oxygen supply planning.

For health technology decision-makers, the issue of oxygen supply is wrought with trade-offs. Systems that meet wide-ranging demand requirements will have positive health benefits but may have high operating costs and oxygen surplus during low demand periods, whereas systems meeting average demand criteria may inadequately account for periods of high variability in demand resulting in adverse health consequences. Our model could thus help determine what strategic mix of both a ‘fixed’ technology system (e.g. concentrators with adequate power supply) meeting some baseline demand, and a contingency backup supply (e.g. additional concentrators, cylinders, or other means of oxygen storage), may be required to cost-effectively meet variability in demand in both low and high seasons. For example, our model could inform decisions about how many concentrators to purchase to meet the majority of expected demand and how much stored oxygen to stock to meet short-term variability beyond the capacity of the concentrators. Model estimates of maximum patient load during high season could inform decisions about the layout of a ward to ensure that adequate access points to oxygen are available to accommodate all patients during busy periods. With estimates of how demand is distributed between low and high seasons, our model could also help inform how to appropriately split budgetary resources between low and high seasons to ensure adequate financial resources are reserved for increased electricity usage, or cylinder refilling, during peak periods.

Our simulation model can be further developed in several areas, particularly with the availability of better data. First, hypoxemia prevalence has been found to be age-dependent, with higher occurrences in neonates compared to older children [37]. Including age variation will be important where age-dependent demand has implications for oxygen technology planning (e.g., different ward locations for neonates, infants and children). Second, hypoxaemia prevalence among pneumonia admissions may be higher during high season, due to increasing severity of illness [37]. The flow rate distribution may also shift to higher levels, corresponding to more severe illness in high season. We lacked sufficient data to model such variations. Third, hypoxaemia is also prevalent in non-pneumonia conditions. For example, high hypoxaemia rates have been found in children with malaria, meningitis, and malnutrition [12]. As more becomes known about hypoxaemia prevalence, our model can easily integrate the compounding effects of multiple illnesses on oxygen demand by simulating separate illness-specific admission streams, each with their own unique admission rate, degree of seasonality, and treatment considerations. By focusing on childhood pneumonia, we are taking a major step towards addressing total paediatric oxygen demand, as it is more likely that pneumonia cases are screened for hypoxaemia and prescribed oxygen. An integrated approach to identifying and treating hypoxaemia in other common childhood illnesses is an issue that deserves serious attention, especially if the oxygen needs of such illnesses are to be adequately met [12]. Lastly, the model could be applied beyond paediatric applications incorporating estimates of oxygen needs for other clinical services (e.g. surgery, adult respiratory illnesses) if data on the five key factors affecting demand represented in the model are available.

The DES model presented demonstrates a novel approach to estimating oxygen demand due to childhood pneumonia in low-resource settings. The model provides a more realistic estimate of time-varying demand at the health facility level, and can be applied to a wide range of geographies by choosing appropriate context-specific inputs. Oxygen is a unique health commodity in that managing the supply can be decentralized to the facility level with the use of local oxygen generating technology. Thus, a better understanding of oxygen need dynamics at the facility level could go a long way in improving health systems planning and cost-effective decision-making around oxygen technologies.

Despite the widespread use of DES in health services research, its application to low-income settings is relatively new. DES can be applied to any health commodity with a temporal demand profile (e.g., rapid malaria diagnostic tests, seasonal vaccines or drug treatments); thus the potential impact of DES as a technique for health resources forecasting is significantly broader than the problem of oxygen.
